# ITS1-PCR based identification of chicken *Eimeria* species in poultry litter from Mymensingh district, Bangladesh

**DOI:** 10.5455/javar.2021.h538

**Published:** 2021-09-20

**Authors:** Mohammad Zahangir Alam, Anita Rani Dey, Shanaz Parvin, Shirin Akter, Sharmin Aqter Rony

**Affiliations:** Department of Parasitology, Faculty of Veterinary Science, Bangladesh Agricultural University, Mymensingh, Bangladesh

**Keywords:** Coccidiosis, *Eimeria* species, identification, ITS1-PCR, chicken

## Abstract

**Objectives:**

The purpose of this study was to determine the species composition of *Eimeria* circulating in Mymensingh district, Bangladesh, using Internal Transcribed Spacer 1 (ITS1) sequences in polymerase chain reaction (PCR) assay.

**Materials and Methods:**

Coccidian oocysts were isolated and sporulated in a solution containing 2% potassium dichromate from litter slurry collected from 13 commercially active broiler farms in the research region. Genomic DNA was isolated from sporulated oocysts and used to amplify the *Eimeria* species-specific *ITS1* gene by PCR amplification. Electrophoresis of 1.5% agarose gel was used to visualize the amplified PCR products.

**Results:**

In the study samples from Mymensingh district, Bangladesh, the presence of *Eimeria brunetti*, *Eimeria acervulina*, *Eimeria necatrix*, *Eimeria mitis*, and *Eimeria tenella* was identified.

**Conclusions:**

The findings of this study may shed light on the zonal approach to chicken coccidiosis control. Additionally, it suggests that ITS1-based PCR might be used in the field to accurately identify *Eimeria* species.

## Introduction

The composition and dynamics of *Eimeria* spp., the causative agent of chicken coccidiosis, are critical factors in determining the severity of the disease [1]. Coccidiosis in hens is one of the most prevalent and economically significant poultry diseases, causing an estimated $13.6 billion USD annual losses [2]. Selective *Eimeria* species infections can be catastrophic and destructive for chicken farms, depending on their prevalence, fecundity, and pathogenicity. Seven *Eimeria* species are widely recognized for infecting chickens with varying degrees of pathogenicity: *E. tenella*, *E. necatrix*, *E. brunetti*, *E. acervulina*, *E. mitis*, *E. maxima*, and *E. praecox* [2,3]. However, the composition of these species varies considerably between geographic locations [4]. The presence of highly pathogenic species, combined with their density, may predispose to coccidiosis epidemics and associated sickness. This infection has the potential to cause significant harm to the host’s gut, hence raising morbidity, mortality, and the risk of subsequent infections [3–5]. Coccidiosis is a disease that is frequently observed in Bangladesh’s chicken farms. As a result, with an average frequency of 34.48% at the bird level [6], chicken coccidiosis appears to be a major constraint on Bangladesh’s poultry output.

Immunity is species-specific, and occasionally strain-specific, and anticoccidial medication resistance are frequently highlighted as common constraints on poultry coccidiosis control worldwide [2,5]. Genetic diversity of the causative agents influences epidemiology, virulence, and their implications for treatment and immunization efforts, as well as coccidiosis control [2]. As a result, it is vital to accurately identify and genotype unique *Eimeria* species for coccidiosis prevention, surveillance, and management. Despite its intraspecies conservation, the Internal Transcribed Spacer 1 (ITS1) gene sequences in eukaryotic cells vary greatly between species [5]. Thus, ITS1 sequences can be utilized as a DNA marker to aid in the identification of a variety of diseases, including *Eimeria* infection. ITS1 primers have made significant progress in a polymerase chain reaction (PCR)-based identification of chicken *Eimeria* species [7]. Numerous investigations have previously employed *Eimeria* species-specific ITS1 primers [7–9]. On the other hand, conventional diagnosis based on oocyst morphology, site-specificity, and the lesion is usually difficult to perform and erroneous [5,8]. As a result, diagnostic laboratories are increasingly relying on DNA-based approaches for *Eimeria* species identification. All seven *Eimeria* species have been successfully identified using PCR-based techniques [2,5].

*Eimeria* spp. has already been identified and characterized molecularly in a number of countries [3,5]. Coproscopy and lesion-based identification of chicken *Eimeria* spp. were conducted in a few areas throughout Bangladesh [10–12]. Regrettably, there still appears to be a dearth of published findings on molecular identification of accessible *Eimeria* species in many other parts of the country. On the other hand, accurate pathogen identification has substantial implications for diagnosis and disease control, biology and epidemiology research, the creation of novel immunogens, and anticoccidial medicine selection [2,3,5,13]. Additionally, the prevalence and distribution of *Eimeria* spp. varied according to geography, evaluation methodology, and host factors such as age, treatment, and population type [13]. For a number of *Eimeria* species, the difference between morphology-based and PCR-based identification was also significant [13]. As a result, the current work used ITS1-PCR to illustrate the spread of *Eimeria* species in broiler farms in Mymensingh.

## Materials and Methods

### Ethical consideration

Prior to the experiment’s start, verbal consent was obtained from each of the farms included in the study. While sampling, animal welfare concerns were reviewed to ensure that routine management processes and avian behaviors were not disrupted.

### Study area and farm selection

The district of Mymensingh in Bangladesh was chosen as the study region due to its accessibility and a large concentration of commercial chicken farms. The farms were chosen based on the ease of obtaining permission to collect sample from the respective farms.’ Litter samples were gathered from 13 independently operated broiler farms. 

### Sample collection and oocyst isolation

Litter sample slurry (about 15–20 liters) was collected from each farm’s adjoining feeder and drinker sites. Using the approach described by Alam et al. [10], litter samples were processed and oocysts were extracted. The microscopic inspection confirmed the presence and concentration of oocysts in the sample. Two percent potassium dichromate was used as culture media to sporulate the concentrated oocysts for 1–2 days at a temperature of 28°C, followed by repeated centrifugation and resuspension in water to remove the media. Finally, the pelleted oocysts were retrieved for DNA extraction.

### Extraction of genomic DNA

Each sample’s pelleted sporulated oocysts were washed once more with a 1 mM sodium hypochlorite solution for 10 min at 4°C, followed by three rinses with deionized water. Using a specialized tissue homogenizer, the sporulated oocysts were broken and crushed. Genomic DNA was isolated according to the manufacturer’s instructions using the QIAamp stool DNA isolation kit (Qiagen, Germany). The extracted DNA samples were measured using a ThermoFisher Scientific Nanodrop 2000 spectrophotometer (USA) and kept at –20°C until further use.

### Amplification of ITS1 of Eimeria spp.

As reported in Table 1, isolated DNA was utilized as a template to amplify the ITS1 region of *Eimeria* spp. To examine the amplification findings, all amplicons were separated on 1.5% agarose gel and then stained with ethidium bromide.

## Results and Discussion

**Figure 1.**
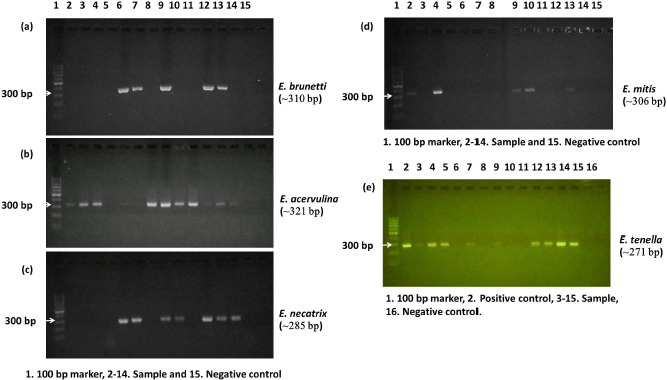
Images showing the Internal transcribed spacer 1 (ITS1)-PCR products from oocyst samples resolved on 1.5% agarose gel. PCR amplification was found using primers specific for (a) *E. brunetti* (~310 bp), (b) *E. acervulina* (~321 bp), (c) *E. necatrix* (~285 bp) and (d) *E. mitis* (~306 bp), and (e) *E. tenella* (~271 bp). DNA ladder of 100 bp molecular weight was used as marker.

Both small and large-scale poultry farming is a well-established sector in Bangladesh, where practically all farms are routinely plagued with coccidiosis [6]. In this country, morphometry-based detection of chicken coccidian protozoa has long been established as the gold standard. Since the last few years, molecular methods have been used to identify parasite species and diagnose parasitic disorders, particularly in research laboratories. *E. brunetti, E. acervulina, E. necatrix, E. mitis,* and *E. tenella* of seven widely available species were identified in litter samples from broiler farms in Mymensingh, Bangladesh, using the ITS1-PCR method (Fig. 1). The concentration and species of chicken *Eimeria* discovered in a few locations around Bangladesh varied significantly between surveys. Iqbal et al. [12] discovered five chicken *Eimeria* species in Bangladesh, including *E. acervulina*, *E. tenella*, *E. necatrix*, *E. maxima*, and *E. brunetti*, based on oocyst shape and host lesion. On the other hand, the abundance of all seven chicken *Eimeria* species was reported based on oocyst morphology in Mymensingh district [10] and partial gene sequences described amplified region marker in the Chittagong district [11]. These enormous variations in data may imply that different diagnostic methods and topographical zones may have an effect on the abundance of *Eimeria* species. 

**Table 1.** Primer sequence of ITS1 for 7 chicken *Eimeria* spp. [8,9].

Species	Primer	Sequence (5′–3′)	Annealing temperature (°C)	Size	Reference
*E. acervulina*	EaF EaR	GGCTTGGATGATGTTTGCTG CGAACGCAATAACACACGCT	60	321	[9]
*E. brunetti*	EbF EbR	GATCAGTTTGAGCAAACCTTCG TGGTCTTCCGTACGTCGGAT	45	310	[9]
*E. maxima*	EmaF EmaR	GTGGGACTGTGGTGATGGGG ACCAGCATGCGCTCACAACCC	65	205	[8]
*E. mitis*	EmiF EmiR	TATTTCCTGTCGTCGTCTCGC GTATGCAAGAGAGAATCGGGA	54	306	[9]
*E. necatrix*	EnF	GTCAGCTTTTGCCTGCCTGGGTG ACAGACCGCTACACAACACG	55	285	[9]
	EnR				
*E. praecox*	EpF EpR	CATCATGGAATGGCTTTTTGA AATAAATAGCGCAAAATTAAGCA	54	368	[9]
*E. tenella*	EtF EtR	AATTTAGTCCATCGCAACCCT CGAGCGCTCTGCATACGACA	60	271	[9]

The possibility of significant discrepancies between microscopy findings and PCR-based identification of *Eimeria* species’ genomic DNA is well reported [14]. This was also evident in the comparison of the current ITS1-PCR-based investigation to our earlier oocyst morphology-based analysis [10], despite the fact that both studies used the same sampling site and litter source for oocyst recovery. In our current investigation, the absence of *E. praecox* or *E. maxima* may indicate a potential error in the morphometric detection method’s categorization of accessible oocysts. Additionally, it underscored the gap between the non-molecular and molecular identification technologies in terms of specificity and sensitivity. ITS1 is considered as a highly species-specific genetic marker for PCR-based chicken *Eimeria* identification due to its highly conserved sequence within species [5]. As a result of this study’s findings, it is clear that classic microscopy or lesion-based techniques are less accurate. The excellent accuracy level of ITS1-PCR assay demonstrates its applicability as a confirmatory field diagnostic method for chicken coccidiosis. The implementation of this molecular tool for the exploration of *Eimeria* species-specific data would permit region-specific strategic control. 

Intracellular *Eimeria* parasites provide a significant economic and welfare risk to the worldwide poultry industry, particularly chickens renowned for their high protein content, rapid generation time, and global acceptability. Due to their high degree of host and location specificity, individual *Eimeria* species can create a variety of host-parasite relationships and cause a variety of clinical or subclinical symptoms in birds [15]. Among the five species identified in our investigation, *E. necatrix* and *E. tenella* have the potential to cause major pathogenic lesions such as severe intestinal bleeding, increased morbidity, and mortality. While *E. acervulina* and *E. brunetti* can cause clinical disease, *E. mitis* is considered a relatively non-virulent para­site [2–5]. They can cause decreased feed conversion ratios and slower growth rates. Notably, despite the presence of parasite DNA in the litter, the absence of clinical disease may imply ineffective coccidiosis control techniques such as chemoprophylaxis or farm management practices [6]. As a result, the economic impact of clinical or subclinical coccidiosis is influenced by the makeup and dynamics of the coccidia population. Additionally, undetected coccidiosis has been linked to anticoccidial resistance or vaccination failure [3,5]. As a result, it is necessary to understand the existing *Eimeria* species profile in order to implement an effective control strategy on the farm.

## Conclusions

As far as the authors are aware, this is the first publication on ITS1-PCR-based identification of *Eimeria* species composition in Mymensingh district, Bangladesh. The presence of highly pathogenic *E. tenella*, *E. necatrix*, and *E. brunetti* in addition to the more prevalent *E. acervulina* and less pathogenic *E. mitis* in the study area indicated that the clinical severity of coccidiosis could be economically devastating, necessitating target-specific therapeutic interventions and vaccination. Additionally, ITS1-based PCR may be advised for reliable diagnosis of chicken coccidiosis on a broad scale to track the epizootiological abundance in Bangladesh.

## List of abbreviations

ITS1, Internal Transcribed Spacer 1; PCR, polymerase chain reaction; DNA, deoxyribonucleic acid; USD, United States Dollar.
